# Histologic Transformation in EGFR-Mutant Lung Adenocarcinomas: Mechanisms and Therapeutic Implications

**DOI:** 10.3390/cancers13184641

**Published:** 2021-09-16

**Authors:** Ranjan Pathak, Victoria M. Villaflor

**Affiliations:** Department of Medical Oncology and Therapeutics Research, City of Hope, Duarte, CA 91010, USA; rpathak@coh.org

**Keywords:** non-small cell lung cancer, epidermal growth factor receptor gene, EGFR, acquired resistance, histologic transformation

## Abstract

**Simple Summary:**

Despite being effective initially, almost all patients eventually develop resistance to EGFR-tyrosine kinase inhibitors (TKI). Changes in the histology of the tumor have been increasingly recognized as a critical mechanism of resistance to EGFR-directed therapies. This article summarizes histologic changes known to impart resistance to EGFR TKIs and discusses novel pathways to develop effective novel therapies.

**Abstract:**

With the advent of potent EGFR tyrosine kinase inhibitors (TKIs), the treatment landscape of EGFR-mutant lung adenocarcinomas has changed drastically in recent years. However, the development of resistance to EGFR TKIs remains a critical barrier to improving survival in these patients. Histologic transformations to small cell lung carcinoma, large cell neuroendocrine carcinoma, squamous cell carcinoma, and the sarcomatoid phenotype have been increasingly recognized as important mechanisms of resistance. In this article, we summarize the known biological bases for such phenotypic switches in regard to EGFR TKIs and describe novel pathways that might be harnessed to develop effective novel therapies for patients with EGFR-mutant non-small cell lung cancers.

## 1. Introduction

With the application of advanced diagnostic techniques, our understanding of the molecular underpinnings of non-small cell lung cancer (NSCLC) has been transformed in the past two decades. Identification of oncogenic driver mutations has ushered in an era of genomic biomarker-directed therapies. Lung adenocarcinomas with activating mutations in the epidermal growth factor receptor (EGFR) gene represent the most common subset of targetable driver-positive non-small cell lung cancers (NSCLCs) and comprise ~10–15% of Caucasian patients and ~40–50% of East-Asian advanced NSCLC patients. There are higher incidences of such cancers in females and those who have either never smoked or are former light smokers [1]. The EGFR gene encodes the EGFR tyrosine kinase, which is a member of the human epidermal growth factor (HER) family of transmembrane receptors (HER1/EGFR, HER2, HER3, and HER4). EGFR tyrosine kinase exists as an inactive monomer that dimerizes with a receptor of the same type or another member of the HER family in response to ligand binding. Activating mutations of the EGFR gene, such as exon 19 deletions or exon 21 L858R point mutations, lead to the constitutional activation of the EGFR gene without ligand-induced stimulation and downstream signaling [2].

In the past few decades, various first (gefitinib and erlotinib) and second-generation (afatinib and dacomitinib) EGFR-tyrosine kinase inhibitors (TKIs) have been developed, which have been shown to be superior to chemotherapy in multiple phase 3 clinical trials, with response rates of ~70–75% and median progression-free survival (PFS) of 10–14 months [3]. These small-molecule TKIs bind the adenosine triphosphate pocket (ATP) of EGFR, thereby inhibiting its autophosphorylation and the resulting downstream signal transduction. Osimertinib, a third-generation EGFR TKI, has been shown to be superior to earlier-generation TKIs in both PFS (18.9 versus 10.2 months) and overall survival (38.6 versus 31.8 months) in a landmark phase 3 randomized clinical trial (FLAURA) [4]. More recently, osimertinib has been shown to be associated with disease-free survival (median was not reached in patients on the osimertinib arm compared with 27.5 months on the placebo arm (hazard ratio, 0.20; 95% confidence interval, 0.15 to 0.27; *p* < 0.0001)) in patients with stage IB to IIIA NSCLCs with completely resected tumors following standard platinum-based adjuvant chemotherapy, setting the scene for further changes in the treatment landscape of EGFR-mutant lung adenocarcinomas [5]. As a result of these advances, EGFR-mutant lung cancer patients are living longer than ever before [6].

**Figure 1:**
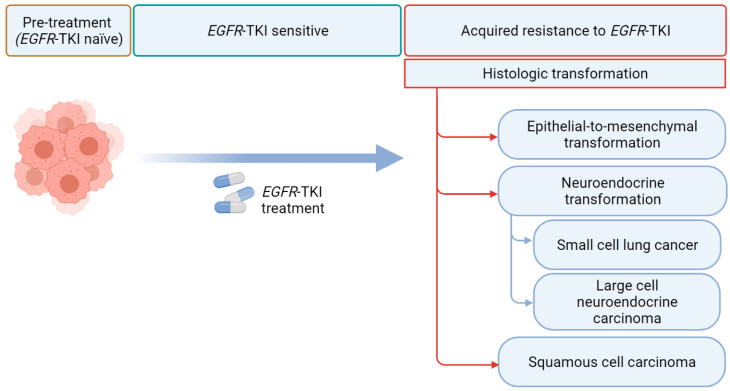
Histological transformation via EGFR tyrosine kinase inhibitors (TKI). EGFR refers to epidermal growth factor receptor (created with Biorender.com).

However, despite being effective, EGFR TKIs eventually lose efficacy in almost all EGFR TKI-treated patients [7]. Drug resistance is thought to arise from the clonal selection pressures exerted by EGFR TKIs with a selection of pre-existing subclones or a selection of randomly acquired alterations that develop over time [8]. Resistance to EGFR TKIs can be broadly classified as innate (defined as disease progression within the first 3 months after TKI initiation) or acquired [9]. Although most acquired resistance develops due to mutations in the EGFR receptor, activation of bypass intracellular signal pathways, or nongenetic adaptive changes, histologic transformation has been increasingly recognized as a crucial resistance mechanism [7,10]. In addition to the phenotypic transformation to small cell lung carcinoma (SCLC) [11,12], multiple recent reports have described transformations to rare histologic phenotypes, such as large cell neuroendocrine carcinomas (LCNECs) and squamous cell carcinomas (SCCs) (Figure 1) [13,14].

In this article, we try to summarize the mechanisms of histologic transformations in EGFR-mutant NSCLCs and discuss potential therapeutic approaches to conquer this important resistance mechanism.

## 2. Targeting EGFR in Lung Cancer

Since the discovery of EGFR signaling in 1962, multiple attempts have been made to target this pathway, including the use of monoclonal antibodies such as cetuximab and panitumumab [15]. Further work in this space led to the identification of EGFR mutations in lung cancer, paving the way to precision medicine in lung cancer [16]. Somatic activating mutations in the EGFR gene result in ligand-independent receptor activation and downstream signaling promoting cell proliferation and survival [2]. The majority of EGFR mutations affect the intracellular tyrosine kinase domain of the receptor and include a heterogeneous group of mutations in exons 18 to 21. Exon 19 deletion and exon 21 L858R point mutations represent the majority of EGFR mutations that are sensitive to EGFR TKIs [17]. Uncommon EGFR mutations such as exon 18 (E709X and G719X), exon 19 (exon 19 insertions), exon 20 (exon 20 insertions and S768I), and exon 21 (L861Q) mutations constitute the remaining 10–15% of EGFR-mutated lung adenocarcinomas, which display variable sensitivities to EGFR TKIs [17].

## 3. Mechanisms of Resistance to EGFR TKIs

Selective pressures from TKI therapy can lead to the selection of cells lacking the original driver mutation or the acquisition of on and off-target resistance mechanisms. These can include the selection of preexisting resistant clones or induced adaptive resistance mechanisms [9]. The on-target (EGFR-dependent) and off-target (EGFR-independent) resistance mechanisms differ significantly according to the specific EGFR TKI used. For instance, patients receiving first or second-generation EGFR TKIs predominantly develop EGFR-dependent resistance mechanisms. Patients receiving the third-generation TKI osimertinib, as either first or second-line therapy, mostly develop off-target resistance, highlighting the differences in resistance mechanisms according to the type of TKI and line of therapy [7,18,19].

### 3.1. EGFR Target-Dependent Mechanisms of Resistance

Changes in EGFR to the critical amino acid residues can preclude the mechanisms of action of EGFR TKIs. The gatekeeper mutation in exon 20 of EGFR with the amino acid substitution of p.Thr790Met results in the steric hindering to the binding of first and second-generation EGFR TKIs to their ATP-binding site on EGFR [20]. Although seen in 50–60% of patients receiving gefitinib, erlotinib, or afatinib, no evidence of T790M mutation was seen to coincide with resistance from plasma genotyping when patients received first-line osimertinib [19]. Since osimertinib is selectively active against both EGFR-sensitizing mutations and the T790M mutations, the lack of emergence of a T790M mutation would not be surprising. With the approval of osimertinib in the first-line setting, the incidence of T790M as a resistance mechanism is expected to become less relevant over time. Although C797X (p.Cys797Ser or p.Cys797Gly) mutations have emerged as the most common EGFR-dependent mechanism of resistance to osimertinib in later lines of therapy, their incidence in the front-line setting is lower (only 7% patients out of 91 patients with paired plasma samples analyzed by next-generation sequencing) in the FLAURA trial [19]. It remains to be seen whether other EGFR-dependent mutations that were described on earlier-generation EGFR TKIs are relevant in the era of first-line osimertinib.

### 3.2. EGFR Target-Independent Mechanisms of Resistance

Continued EGFR TKI exposure can lead to rewiring of the non-EGFR cell signaling pathways that can lead to EGFR TKI resistance. Mesenchymal epithelial transition factor (MET) gene amplification has been reported in 5–22% of patients experiencing disease progressing in spite of first-generation EGFR TKIs [7,21]. Despite the challenges in harmonizing the definitions of MET amplification and the lack of consensus on the definition of MET amplification using liquid biopsies, MET receptor tyrosine kinase signaling is thought to be the most commonly altered pathway involved in EGFR TKI resistance, irrespective of the EGFR TKI used or the line of therapy [22]. HER2 amplification, PIK3CA, and rat sarcoma (RAS) mutations have also been described as possible mechanisms of resistance in liquid biopsy-based analyses from the FLAURA trial [19]. In addition to these mutations, numerous fusions/chromosomal rearrangements (such as the rearranged during transfection (RET), neurotrophic tropomyosin receptor kinase (NTRK), and ROS1), mutations in cell cycle-related genes (such as cyclin-dependent kinases 4 and 6, and cyclin-dependent kinase inhibitor 2A (CDKN2A)) have also been described as possible EGFR target-independent mechanisms of resistance to EGFR TKIs [22]. With the introduction of first-line osimertinib, further work is needed to understand the relative incidences of various types of EGFR-independent mechanisms of resistance in the Osimertinib era.

### 3.3. Histologic and Phenotypic Transformations

Histologic transformations in EGFR TKI treated patients have emerged as important mechanisms of resistance to EGFR TKIs. Although SCLC transformation has been considered to be the most common histologic transformation in EGFR-mutant NSCLCs in patients with disease progression during treatment with an earlier-generation EGFR TKI, [7] SCC transformation emerged as a potentially more common (15%) histologic transformation in front-line osimertinib-treated patients in a small cohort of patients with limited follow-up [18]. Transformed tumors generally retain the initial EGFR mutations [12,14]. An essential consideration in the diagnosis of histologic transformation is the possibility of preexisting initial mixed histology, which often cannot be precisely assessed on small biopsies/cytologic samples, highlighting the challenges of studying histologic transformations. Unfortunately, the current treatments for patients with histologic transformations are primarily driven by histology; further understanding of the biology and the mechanisms of histologic transformation are urgently needed to develop precision medicine for these patients.

## 4. Histologic Transformations in EGFR TKIs Treated NSCLCs

### 4.1. Epithelial-to-Mesenchymal Transformation

Epithelial-to-mesenchymal transformation (EMT) refers to the process of epithelial cells transforming to mesenchymal cells by losing their polarity and intercellular adhesions [23]. “Sarcomatous/sarcomatoid dedifferentiation” is another term that is used by pathologists in clinical practice to imply EMT [24]. These changes are accompanied by anti-apoptotic signals and changes in molecular expression (downregulation of epithelial cytokeratin markers and upregulation of mesenchymal markers such as vimentin and fibronectin) [25]. The culmination of these changes imparts cancer cells with invasiveness, metastatic potential, and drug resistance. Thus, such changes mean poor prognoses for multiple types of cancer, including NSCLC [26,27].

Although some preclinical models have suggested EMT as a mechanism of acquired resistance to EGFR TKIs, [28] further work is needed to understand whether EMT is truly a driver of resistance, as opposed to a passenger event [29]. Early reports describing EMT imparting acquired resistance to EGFR TKIs, such as those by Uramoto et al. and Sequist et al., were based on the absence of any other mechanisms of resistance known at that time, and therefore may have overestimated the impact of EMT. Moreover, since early studies examining the associations of EMT signatures and sensitivity with EGFR TKIs were carried out in unselected patients irrespective of sensitizing EGFR mutations, [30] it is unclear whether those signatures remain valid for EGFR-mutant NSCLCs.

Although a relationship between sensitivity to EGFR TKIs and tumor E-cadherin expression has been proposed, depletion of cadherin 1 (CDH1) by itself in EGFR-mutant therapy-naïve NSCLC cells does not lead to a loss of EGFR TKI efficacy [31]. These observations suggest that both the downregulation of epithelial markers (that are repressed by several nuclear factors, such as zinc finger E-box binding homeobox 1 (ZEB1) (δEF1, ZFHX1A), ZEB2 (SIP1, ZFHX1B), snail family transcriptional repressor 1 (SNAI1) (Snail), SNAI2 (Slug), E12/E47, and TWIST) and upregulation of mesenchymal proteins are necessary to impart resistance to EGFR TKIs [32]. Preclinical studies have also revealed that the pro-EMT factor TWIST leads to inhibition of pro-apoptotic protein BCL2-interacting mediator of cell death (BIM) either by directly binding to its promoter or by inducing ZEB1 in EGFR-mutant NSCLC murine models [28,33]. EMT may, therefore, induce EGFR TKI resistance, at least in part, via transcriptional suppression of BIM-mediated apoptosis. Finally, silencing of microRNAs (such as miR-200) through promoter methylation has been shown to play a pivotal role in the development of EMT [34,35,36]. Ectopic expression of CRIPTO1 (an oncofetal, membrane-associated protein of the epidermal growth factor (EGF) cryptic family 1 gene (CFC) family) upregulated ZEB1 and activated the SRC pathway via microRNA-205 (miR-205) downregulation, thereby promoting EMT and erlotinib-resistance in EGFR-mutant NSCLC cell lines [37]. Anexelekto (AXL)-signaling has been associated with acquired EGFR TKI resistance through the induction of EMT [38]. Cancer-associated fibroblasts (CAFs) have been implicated in the induction of EMT via paracrine mechanisms, which might allow novel approaches to prevent the development of EMTs [39]. Recently, hypoxia has been shown to activate insulin-like growth factor 1 receptor (IGF1R), which can lead to the generation of primitive cancer stem cells and EMT [40].

#### Novel Therapies

EMT is believed to be associated with a reversible state of reduced drug sensitivity that precedes the onset of terminal resistance [41]. Drug tolerant persisters are associated with an altered chromatin state with high expression levels of lysine demethylase 5A (KDM5A), a histone demethylase H3 Lys 4 (H3K4), and other chromatin-modifying enzymes. Epigenetic therapies, such as histone deacetylase (HDAC) inhibitors, have been evaluated and shown to restore the expression of CDH1 and sensitivity to EGFR TKIs in preclinical models [41,42]. Trials combining epigenetic therapies such as HDAC inhibitors with EGFR TKIs in patients developing EMT are needed to test these hypotheses. In addition to epigenetic therapies, 1,25(OH)_2_D_3_-based combination therapies are currently being evaluated in preclinical models for EMT-associated EGFR TKI resistance [29]. Studies also suggest inhibition of BIM by the pro-EMT factor TWIST [28,33]. Hence, B-cell lymphoma 2 (BCL-2) homology 3 (BH3) mimetics, which target the B-cell lymphoma (BCL) family of proteins and leverage the apoptotic pathway, have been proposed as a means of overcoming EMT-associated EGFR TKI resistance [28,33].

Other approaches to reverse EMT-mediated EGFR TKI resistance include targeting specific bypass mutations (such as mitogen-activated protein kinase (MAPK) and fibroblast growth factor receptor 1 (FGFR1)), microRNAs [36], immune checkpoint inhibitors, [43] AXL receptor tyrosine kinase, [38] IGFR1, [40] etc. Targeting EGFR and SRC tyrosine kinase reduced the growth of CRIPTO1-positive, erlotinib-resistant, EGFR-mutant NSCLC cells, suggesting that this combination might be able to counteract intrinsic resistance to EGFR-TKIs in patients with CRIPTO1-positive, EGFR-mutant NSCLC undergoing EMT [37]. Pharmacological inhibition of neurogenic locus notch homolog protein 1 (NOTCH1) signaling pathway is currently being evaluated as a means of restoring sensitivity to EGFR TKIs (NCT01158404, NCT01653470). Finally, further understanding of the role of the MET pathway in EMT could pave the way towards the therapeutic application of MET inhibitors in EMT-driven resistant patients (Table 1) [44].

### 4.2. Neuroendocrine Transformation

Although SCLC transformation has been well described as a mechanism of acquired resistance in EGFR-mutant NSCLCs, several recent reports have also identified LCNEC as a means of resistance to EGFR TKIs.

#### 4.2.1. SCLC

Studies have estimated that ~5–15% of EGFR-mutated lung adenocarcinomas treated with EGFR TKIs transform to SCLCs [7,10]. Patients with SCLC transformation tend to have poorer prognoses [12,45]. Preclinical studies in mice and cell lines have shown that RB1 inactivation is essential for SCLC development [46,47]. This observation has been further reinforced by the sequencing of human SCLCs showing mutation in or loss of retinoblastoma gene (RB1) in 100% of samples [48]. However, RB1 is necessary but not sufficient for SCLC development from adenocarcinomas. Additional pathways such as NOTCH-Achaete-scute homolog 1 (ASCL1) and TP53 perhaps play essential roles [49].

In a study aimed at characterizing the molecular changes in transformed SCLCs, Niederst et al. observed that transformed SCLCs share many features of classical SCLCs, such as universal loss tumor suppression via RB1 and TP53 inactivation, reduced or absent EGFR protein expression, epigenetic changes, and increased sensitivity to BCL-2 inhibition. All patients with transformed SCLCs harbored the original activating EGFR mutation [50]. Kim et al. showed that the divergence of SCLC clones from adenocarcinomas occurred early on, even before the initiation of EGFR TKIs. The authors also found that complete inactivation of both RB1 and TP53 increased the risk of SCLC transformation by >42 fold [51]. Apolipoprotein B mRNA editing enzyme, catalytic polypeptide-like (APOBEC)-induced hypermutation was frequent in the branches toward SCLC transformation [51]. The loss of sensitivity to EGFR TKIs following SCLC transformation seems to be mediated partly by the downregulation of the expression of EGFR protein, although alterations in EGFR-dependent and EGFR-independent pathways might also contribute [52]. This has been reflected in clinical experience from two large case series. Ferrer et al. reported a multicenter retrospective study evaluating clinical outcomes of SCLC transformation in 48 EGFR mutant NSCLCs between 2005 and 2017 [11]. The median time to SCLC transformation was 16 months. The median overall survival after transformation was only nine months, which mirrors survival seen in de novo extensive-stage SCLC patients. Marcoux et al. reported another multicenter retrospective study evaluating clinical outcomes of 58 patients with transformed SCLC from adenocarcinomas [12]. The median time to transformation was similar to the Ferrer et al. series. A few responses were seen in patients who had concurrently active NSCLC clones. Median survival after transformation was 10.9 months [12].

Data implicating RB1 loss as a driver of SCLC transformation is further supported by Offin et al., who found that “triple-positive” patients with EGFR/RB1/TP53 mutations had an at least 6-fold higher risk of SCLC transformation compared to EGFR-mutant lung adenocarcinomas without RB1/TP53 mutations. The authors also noted that triple-mutant tumors had enrichment of whole-genome doubling and an APOBEC signature, which may represent early genomic determinants of lineage plasticity [53]. However, not all EGFR/ RB1/TP53 triple-mutant NSCLCs undergo SCLC transformation, highlighting the importance of additional pathways such as epigenetic changes [29]. These data suggest that multiple genetic hits are probably needed to transform adenocarcinomas to SCLCs, which has made the development of pre-clinical models of transformed SCLCs challenging.

Although biopsy is essential to establishing the diagnosis of SCLC transformation, serial elevations of blood neuron-specific enolase (NSE) or pro-gastrin-releasing peptide (GRP) might help detect the emergence of SCLC clones earlier before they manifest clinically [54]. Serial rises in pro-GRP and NSE levels have been noted to mirror SCLC transformation in numerous case reports and deserve further studies to determine their clinical utility in patients EGFR TKI-treated NSCLCs [55,56,57].

##### Novel Therapies

Various strategies to overcome acquired resistance due to SCLC transformation are currently being investigated. The combination of EGFR TKIs with upfront platinum-etoposide chemotherapy is being evaluated in patients with triple-mutant cancers at high risk of transformation (NCT03567642). In addition to cytotoxic chemotherapy, approaches utilizing BCL-2 family inhibitors may be an attractive target in transformed SCLC patients [50]. Targeting cell cycle vulnerabilities created due to RB1 loss, such as sensitivity to checkpoint kinase 1 (CHK1) and polo-like kinase 1 (PLK1) inhibitors (which target DNA damage checkpoints), might yield synthetic lethality in transformed SCLCs [58]. Similarly, dependence on aurora kinases (AURK) arising from RB1 loss [59] (coupled with AURKA mediated acquired resistance to EGFR TKIs in RB1 proficient clones [60]) may open doors to combination therapies with aurora kinase inhibitors, e.g., a phase 1/1b clinical trial of AURKA inhibitor alisertib with osimertinib in metastatic EGFR-mutant lung cancer (NCT04085315). Modulation of epigenetic pathways (such as enhancer of zeste 2 polycomb repressive complex 2 subunit (EZH2)) has been proposed as a means of not only treating patients with transformed SCLC but also preventing SCLC transformation. EZH2 inhibitors have been hypothesized to prevent SCLC transformation in high-risk triple-mutant cancers, based on SCLC data and small cell transformation in other cancers [29,47,61]. Further studies which examine signaling pathways in transformed SCLCs might pave the way to novel targets in future.

#### 4.2.2. LCNEC

Although LCNECs share many morphologic, phenotypic, and genetic features of SCLC, they also differ in many important ways [62,63]. Only a handful of LCNEC transformations have been reported in EGFR-mutant NSCLCs, thereby limiting our understanding of the mechanisms of this phenotypic change [13,64,65]. Case reports have suggested that in some cases, subclonal cell populations harboring molecular markers of neuroendocrine differentiation such as TP53 and RB1 loss could be present at baseline, which may be selected under the EGFR TKI treatment pressure leading to pathologically evident transformation in due course [64]. Further research is needed to understand the biology and mechanisms of LCNEC transformation in EGFR-mutant NSCLCs.

### 4.3. SCC Transformation

Adenocarcinoma to SCC lineage transformation is the least common type of acquired resistance to EGFR TKIs, although the incidence of SCC transformation during first-line osimertinib therapy might be higher than previously seen with later-line osimertinib or earlier-generation EGFR TKIs [18,66]. Although multiple case reports have suggested resistance to EGFR TKIs after developing SCC transformation, few case reports have suggested durable responses to TKIs, highlighting that SCC transformation may not always contribute to EGFR TKI resistance [14,67]. Patients with de-novo EGFR-mutated SCC tend to have a shorter PFS than their adenocarcinoma counterparts [68]. However, the biology of SCC transformation remains poorly defined, and it is not uncommon for NSCLCs to have coexistent adenocarcinoma and squamous cell carcinoma components [69]. Therefore, squamous “transformation” after treatment could reflect a shift in the predominant histology of a mixed tumor rather than a true lineage shift (as could be the case with other histologic transformations)—especially in cases of small biopsy/cytologic samples where histology may not have been accurately assessed.

Higher frequencies of mutations in neurofibromatosis (NF1), ataxia telangiectasia and Rad3 related (ATR), and breast cancer (BRCA1) genes have been reported in EGFR-mutant SCCs compared to EGFR-mutant adenocarcinomas [68]. However, despite comprehensive genomic analyses, no consistent molecular signature has been identified in SCC transformation; considerable genomic complexity is seen in these patients with mutations in phosphoinositide 3-kinase (PI3K)/AKT/mechanistic target of rapamycin (mTOR) pathways, chromosome 31 amplification, and FGF amplification [66]. In a study examining the acquired resistance mechanisms to osimertinib using paired pre and posttreatment tissue samples, patients with SCC transformation did not have any overarching genomic correlate; patients had the PIK3CA E726K mutation, chromosome 3q gain (and FGF amplification), and PIK3CA copy number gains [18]. Genetically engineered mouse models have shown that the loss of liver kinase B1 (LKB1, also known as serine/threonine kinase (STK11)) can lead to lineage plasticity and SCC transformation in Kirsten rat sarcoma 2 viral oncogene homolog (KRAS) mutant lung cancers [70]. However, concurrent LKB1 loss is rare in human EGFR-mutant NSCLCs [71,72]. Further studies are therefore needed to understand the gene expression of the transformed cases and to assess the nongenomic processes, such as transcription factor and epigenome changes, to determine the biological basis of SCC transformation in EGFR-mutant NSCLCs.

#### Novel Therapies

Therapies targeting the PI3K/AKT/mTOR pathway need to be studied systematically in the setting of clinical trials. Further work is urgently needed to understand novel pathways in transformed SCC, including the epigenetic pathways which may help discover new therapies for these patients [66].

## 5. Conclusions and Future Directions

Improved understanding of the biology and molecular underpinnings of histologic transformation in EGFR-mutated NSCLCs is urgently needed to help develop reliable biomarkers and effective novel therapies. Although EGFR/RB1/TP53 triple mutation seems to identify a group of patients at the highest risk for SCLC transformation, biomarkers to predict transformation to SCC, EMT, and LCNEC are currently lacking. Studies incorporating mid-treatment serial biopsies might help identify clones that drive eventual resistance: the information gained from evaluating these clones might identify potential biomarkers. Bioinformatics and computational biology techniques might enable the development of novel multiomics biomarkers that can identify patients at risk of transformation during early, tailored combination therapies can then be introduced early to eradicate the seeds of resistance before they manifest clinically.

Identification of epigenetic alterations and novel pathways seen with the transformed phenotypes (such as FGFR for EMT or AURKA for RB1-deficient, transformed SCLC) can help develop novel therapies. Some of these therapies might be applied early to block lineage plasticity and prevent the development of abnormal phenotypes or allow restoration of EGFR dependency and EGFR TKI sensitivity once transformation emerges. In addition, multimodality approaches such as the early introduction of stereotactic ablative radiotherapy of metastatic sites should be evaluated to prevent the development of transformed clones in patients deemed high risk for histologic transformation. Multi-arm trials such as the ORCHARD trial (NCT03944772) might provide a unique opportunity to explore treatment options for patients with SCC and SCLC transformation through histology-driven cohorts.

Blood-based tumor analysis offers a non-invasive and easily accessible modality for cancer diagnostics [73]. Despite the rising popularity of circulating tumor DNA (ctDNA) based testing, clinicians should be mindful that histological transformation cannot be detected by blood-based testing alone. ctDNA at the time of progression, however, might help identify patients with RB1 and TP53 alterations, who are at higher risk of SCLC transformation and will benefit from further investigation of the tissue. Histologic and ctDNA analyses should be used as complementary modalities to further our understanding of histologic transformation in EGFR-mutated NSCLC patients. Dynamic monitoring of ctDNA offers an attractive tool to investigate the emergence of resistance mutations and subclonal events. This approach in clinical practice is being evaluated in the APPLE trial, which aims to compare the initiation of the treatment of EGFR^T790M^ based on ctDNA against radiological evidence of disease progression [74]. Although data on osimertinib are still evolving, histologic transformation has been seen in up to 15% of osimertinib-treated patients in early analyses [18]. Further investigation is, therefore, urgently needed to understand the mechanisms of histologic transformation in response to newer-generation EGFR TKIs, such as osimertinib. Finally, further work is needed to understand the incidence of histologic transformation in patients receiving adjuvant osimertinib who only have microscopic disease.

In conclusion, histologic transformation is an emerging and important mechanism of resistance to EGFR TKIs with limited therapeutic options. Although the overall survival of EGFR-mutant NSCLC patients has improved in recent decades, much work needs to be done to understand various mechanisms of resistance so that effective therapies can be developed. Serial mid-treatment biopsies of tumor tissue and ctDNA at baseline and progression to detect histologic transformation should be incorporated in all future clinical trials with EGFR TKIs, whenever possible. Further studies of signaling pathways in tumors with histologic transformation will hopefully help elucidate novel mechanisms that can help develop new biomarkers and discover new therapies.

## Figures and Tables

**Table 1:** Novel pathways and potential therapies for histologically transformed EGFR-mutant lung cancers.

Histologic Type	Novel Pathways and Potential Therapeutics
Small cell lung cancer	▪ Cytotoxic chemotherapy (platinum-etoposide) for triple-mutant cancers at high risk of transformation ▪ BCL-2 inhibitors to target apoptosis resistance ▪ CHK1, PLK1, and AURKA inhibitors to exert synthetic lethality arising from RB1 loss ▪ EZH2 inhibitors to modulate epigenetic pathways
Squamous cell cancer	▪ Targeted therapies to overcome dependencies on PI3K/AKT/mTOR pathways
Epithelial-to-mesenchymal transformation	▪ HDAC inhibitors, 1,25(OH)_2_D_3-_based combination therapies to modulate epigenetic pathways ▪ BCL inhibitors to overcome apoptosis resistance ▪ Targeted therapies to overcome dependencies on alternative pathways such as MAPK, FGFR1, MET ▪ Targeted therapies against NOTCH signaling pathways, AXL receptor tyrosine kinase, IGFR1

AXL = anexelekto; AURKA = aurora kinase A; BCL = B-cell lymphoma 2; CHK1 = checkpoint kinase 1; EGFR = epidermal growth factor receptor; EZH2 = enhancer Of zeste 2 polycomb repressive complex 2 subunit; FGFR = fibroblast growth factor receptor 1; HDAC = histone deacetylase; IGF1R = insulin-like growth factor 1 receptor; MAPK = mitogen-activated protein kinase; MET = mesenchymal epithelial transition factor; NOTCH1 = neurogenic locus notch homolog protein 1; PI3K/AKT/mTOR = phosphoinositide 3-kinase (PI3K)/AKT/mechanistic target of rapamycin; PLK1 = polo-like kinase 1; Rb = retinoblastoma.
